# Association between endometriosis and sexual satisfaction among a sample of lebanese women

**DOI:** 10.1186/s12905-023-02323-1

**Published:** 2023-04-06

**Authors:** Maya Kfoury, Habib Barakat, Souheil Hallit, Sahar Saliba

**Affiliations:** 1 School of Arts and Sciences, Holy Spirit University of Kaslik, P.O. Box 446, Jounieh, Lebanon; 2 School of Medicine and Medical Sciences, Holy Spirit University of Kaslik, P.O. Box 446, Jounieh, Lebanon; 3 Department of Obstetrics and Gynecology, Notre Dame des Secours University Hospital Center, Street 93, Postal Code 3, Byblos, Lebanon; 4 Applied Science Research Center, Applied Science Private University, Amman, Jordan; 5 Research Department, Psychiatric Hospital of the Cross, Jal Eddib, Lebanon

**Keywords:** Endometriosis, Sexual satisfaction, Couple satisfaction, Psychological distress

## Abstract

**Background:**

Endometriosis is a complex disease that affects approximately 10% of women of childbearing age. It is characterized by the presence of endometrial-like tissue outside the uterus and often results in symptoms such as pelvic pain and infertility. This condition may disturb mental health and decrease both the mental and physical health related quality of life of women. The aim of this study was to assess the emotional state and the intimate relational aspect of life in Lebanese women living with endometriosis.

**Methods:**

This is a case-control study that included 317 women (65 participants with endometriosis and 252 controls (1:3 ratio)). Participants completed an online survey that included questions related to endometriosis, Sexual Satisfaction Scale for Women, Couple Satisfaction Index, Perceived Partner Responsiveness Scale and the Depression, Anxiety and Stress Scale.

**Results:**

A significantly higher mean sexual satisfaction score was found in women without endometriosis compared to those with endometriosis (90.83 vs. 83.42; p = 0.006). No significant difference was found in terms of couple satisfaction, depression, anxiety, stress, and perceived partner responsiveness between the two groups. Higher couple satisfaction (Beta = 1.30) and perceived partner responsiveness (Beta = 0.16) were significantly associated with higher sexual satisfaction, whereas higher depression (Beta=-1.70) and having endometriosis compared to not (Beta=-6.51) were significantly associated with lower sexual satisfaction.

**Conclusion:**

This study validated the link between endometriosis and sexual satisfaction and invalidated the association between endometriosis and emotional state, as well as couple satisfaction and perceived partner responsiveness. Greater sexual satisfaction was, however, linked to greater perceived partner responsiveness and greater couple satisfaction, as well as decreased signs of depression, anxiety, and stress. More research is warranted to better understand how Lebanese women are coping with the disease and how clinicians may help them further.

## Background

Endometriosis is a common disease that affects approximately 10% of women and girls of reproductive age worldwide according to the World Health Organization [1]. This disease is mainly associated with pelvic pain and infertility and is characterized by the presence of endometrial-like tissue outside the uterus [2]. These tissues can cause a chronic inflammatory reaction that can lead to the formation of scar tissue in the pelvic area, as well as other parts of the body [3]. Clinically, endometriosis is associated with a wide range of physical symptoms, such as severe menstrual pain, painful intercourse, chronic pelvic pain, excessive bleeding, dyschezia, dysuria, abdominal bloating, nausea, fatigue, and infertility [2]. Moreover, the psychological distress associated with the condition cannot be overlooked. Strong correlations between symptoms of endometriosis and several forms of emotional distress, including depression and anxiety, have been found. Additionally, although several researchers have demonstrated that endometriosis impairs the health-related quality of life and work productivity of women from diverse countries and ethnicities, women continue to experience delayed diagnosis of endometriosis in primary care as the varied and widespread symptoms of the disease result in difficulties in diagnosing it [4]. The normalization of symptoms of pain among young women also leads to greater delays between the onset of symptoms and the diagnosis [5].

In addition to the previously mentioned challenges that women with endometriosis may face, symptoms can affect different aspects of a woman’s life, including the sexual aspect, and are consequently associated with higher levels of relationship distress [6]. In fact, studies suggest that endometriosis symptoms may affect intimate and sexual relationships [7]. Sexuality incorporates psychosocial and physiological factors affecting not only physical health, but also psychological well-being and relationships. Dyspareunia, a common symptom of endometriosis, has been shown to be associated with reduced levels of sexual desire and arousal, and lower intercourse frequency [8]. Recently, there has been a growing interest around the world to study the impacts of endometriosis on women. Research suggests that patients with endometriosis are often affected in terms of infertility and pain at the physical level and in terms of intimate relationships and sexual intimacy at the social level. At a psychological level, these women are likely to experience heightened anxiety and stress [9].

For women with endometriosis, partner support is very important and essential; women who perceive their partner as knowledgeable and interested in their state of health are likely to show greater relationship satisfaction and dyadic coping [7].

When exploring sexual well-being in couples with genito-pelvic pain, perceived partner responsiveness was found to be an important indicator; when both women and partners reported greater perceived responsiveness of the partner, women reported better sexual function and satisfaction and partners reported better sexual function [10]. Responsiveness is an important part of effective communication in relationships. Women diagnosed with sexual dysfunction express concern about recurring themes in the sexual satisfaction literature.

Regarding previous studies, articles focusing on the subject of endometriosis in Lebanon are very few. Through the literature, we could identify only one study that addressed the topic of mental health in patients with endometriosis and that included a Lebanese population [11]. The study found correlations between endometriosis status and psychological disorders (anxiety and depression) [11]. Infertility has been shown to affect women’s well-being, as well as their marital life. Additionally, literature suggests that Arab women may be particularly affected, as women are culturally often blamed and held responsible in Arab countries when it comes to pregnancy and conception issues [11]. In Lebanon, the sexual self of women seems to be often shaped by the needs of men. Women tend to sacrifice their own sexual desires and needs in an attempt to keep their marital and family life together, whereas others are challenging these traditional attitudes [12]. When it comes to sexual difficulties, the decision to seek help still largely depends on the husband, since the latter is generally considered the first and most important support for Lebanese women [13]. Other factors that influence help-seeking decisions may include the accessibility, availability, and quality of sexuality-related care [13].

No other studies have aimed to assess sexual satisfaction among women with endometriosis, as well as perceived partner responsiveness and couple satisfaction simultaneously. The aim of this study would be to (1) compare women with and without endometriosis in terms of sexual satisfaction, perceived partner responsiveness, psychological distress and couple satisfaction, and (2) evaluate correlates of sexual satisfaction among those women.

## Methods

### Study design and participants

A total of 317 Lebanese women participated in this case-control study conducted in May - August 2022, 65 of them with endometriosis and 252 without endometriosis (ratio 1:3). Cases were recruited from gynecologists clinics, whereas controls were recruited in a convenient way using the snowball technique; a Google form was designed and distributed via social networks to women in all Lebanese areas. The study link was disseminated via social media where participants were asked to forward this link to others they know. The purpose of the study was explained in the consent form. The anonymity of participants was ensured. All registrants were free to accept or decline the invitation, with no monetary reward received in return. Participants were also informed that they could withdraw at any time. Participants were all Lebanese women, over the age of 18, and had been sexually active in the past 12 months. Women with chronic pain unrelated to endometriosis, women with menstrual disorders or other gynecological symptoms/syndromes unrelated to endometriosis, women with infertility unrelated to endometriosis, women who have reached menopause, and pregnant women at the time of the study were all excluded; 407 women in total agreed to participate, 317 of whom met our inclusion criteria. All 317 participants answered the socio demographic questions then were asked whether they had been previously diagnosed with endometriosis. As cases and controls were selected through a public survey, there was no match for age and education.

### Calculation of minimum sample size

For sample size calculation, the expected difference in total SSSW score between women with endometriosis and a control population was chosen as the primary outcome. We considered a difference of 0.5 standard deviations to be clinically relevant. Under the assumption of normality, it was calculated that with an alpha of 0.05 and a beta of 0.20, a sample size of 64 women was needed in the case group and a ratio of 1:3 necessary [14]. The minimum sample required in this study was then 64 women for each group. We were able to collect data from 65 women with a diagnosis of endometriosis and 252 women without a diagnosis.

### Questionnaire and measuring instruments

The digital questionnaire was created on Google forms in the native language of Lebanon (Arabic) and took approximately 10 min to complete. The questionnaire was divided into the following sections:

- Sociodemographic questions (Marital status, age, educational level, household crowding index calculated by dividing the number of persons by the number of rooms in the house except the kitchen and the bathroom [15]. Participants were also asked to self-report the financial burden on a scale from 1 = Lowest financial burden to 10 = Highest financial burden.
- Questions related to the disease (diagnosis, symptoms, treatment etc.).
- Perceived Partner Responsiveness Scale (PPRS): The Perceived Partner Responsiveness Scale is a scale designed to assess people’s perceptions of their partner’s responsiveness to them in terms of two properties, understanding and validation [16]. A self-report instrument of 16 items, designed to assess people’s perceptions of their partner’s responsiveness to them. The scale defines responsiveness in terms of two properties, understanding (8 items) and validation (8 items). After reading each item, participants are asked to indicate the degree to which she agrees or disagrees (1: not true at all, 3: somewhat true, 5: moderately true, 7: very true, 9: Completely true). Higher scores indicate higher perceived partner’s responsiveness. The PPRS has been shown to have excellent psychometric properties and has demonstrated its effectiveness as a research tool and will be used in our study to assess perceived partner responsiveness by women with endometriosis [16]. (McDonald’s omega in this study = 0.96)
- Sexual Satisfaction Scale for Women (SSS-W): a scale that assesses sexual satisfaction and sexual distress in women through 30 items scored following different answers options. This scale exhibits strong psychometric properties and has a demonstrated ability to discriminate between clinical and non-clinical samples, and it is composed of five domains supported by factor analyses: contentment, communication, compatibility, relational concern and personal concern. Higher scores indicate higher sexual satisfaction [17]. (McDonald’s omega in this study = 0.95)
- Couple Satisfaction Index (CSI-4): a psychometrically optimized 4-item self-report scale assessing relationship satisfaction. CSI-4 scores can range from 0 to 21. Higher scores indicate higher levels of relationship satisfaction. CSI-4 scores below 13.5 suggest notable relationship dissatisfaction [18]. (McDonald’s omega in this study = 0.91)
- Depression Anxiety Stress Scale (DASS-8): A set of three self-report scales designed to measure emotional states of depression, anxiety, and stress. Each of the DASS-8 depression and anxiety subscales contains 3 items, and the stress subscale contains 2 items (total of 8 items). Depression, anxiety and stress scores are calculated by summing the relevant item scores. Higher scores indicate higher levels of Depression, anxiety and stress [19]. In our study, the DASS − 8 was used to assess depression, anxiety and stress among Lebanese women with endometriosis in order to examine the emotional state. The McDonald’s omega values in this study were as follows: depression = 0.85, anxiety = 0.81 and stress = 0.74.

### Translation process

Since the PPRS and SSS-W scales are not validated in Arabic, they have been translated from English to Arabic (back and forth translation). The first translation from English to Arabic was done by a bilingual translator, familiar with the terminology of the scales, whose native language is Arabic and is fluent in English. A reverse translation was then carried out by a bilingual translator familiar with the concepts of the scales as well. A pilot test was done on 20 women to check if all items were understood. No changes to the scales were done afterwards.

### Statistical analysis

The SPSS software v.22 was used for the statistical analysis. All scores (sexual satisfaction, couple satisfaction, depression, anxiety, stress, and perceived partner responsiveness) were considered normally distributed since the skewness and kurtosis values varied between − 2 and + 2 [20]. Student t test was used to compare two means, whereas Pearson test was used to correlate two continuous variables. A linear regression taking the sexual satisfaction score as the dependent variable was conducted; variables that showed a p < 0.25 in the bivariate analysis were taken as independent ones in the final model. Multicollinearity between variables was verified via the calculation of the variance inflated factor (VIF) < 10 [20]. P < 0.05 was considered statistically significant.

## Results

A total of 317 participants completed the survey; their mean age was 29.46 ± 6.97 years, with 20.5% of them having endometriosis. Other characteristics of the sample are summarized in Table 1. Moreover, 80% of the women declared that pain was the motivation for the physician’s consultation when diagnosed with endometriosis. The main symptoms of endometriosis experienced by women were pain especially in the lower back (81.5%), pain especially excessive menstrual cramps that can be felt (73.8%) and abnormal of heavy menstruation (70.8%) (Table 2).

**Table 1:** Sociodemographic and other characteristics of the participants (N = 317)

Variable	N (%)
Marital status	
Single / divorced	155 (48.9%)
Married	162 (51.1%)
Education level	
econdary or less	30 (9.5%)
University	287 (90.5%)
Work status	
Unemployed	118 (37.2%)
Employed	199 (62.8%)
Have children	
No	202 (63.7%)
Yes	115 (36.3%)
Diagnosed with endometriosis	
No	252 (79.5%)
Yes	65 (20.5%)
	**Mean ± SD**
Age (in years)	29.46 ± 6.97
Household crowding index (person/room)	1.03 ± 0.55
Financial burden	5.83 ± 2.48
Average number of comorbidities	0.70 ± 1.17
Couple satisfaction	14.78 ± 4.58
Depression	4.59 ± 3.02
Anxiety	4.15 ± 2.93
Stress	3.80 ± 1.92
Perceived partner responsiveness	124.09 ± 31.68
Sexual satisfaction	89.31 ± 19.43

**Table 2:** Characteristics of the participants with endometriosis (N = 65)

Variable	N (%)
Motivation for consultation when diagnosed with endometriosis	
Difficulty in getting pregnant	19 (29.2%)
Pain	52 (80.0%)
Family history	10 (15.4%)
Symptoms of endometriosis	
Pain especially lower back	53 (81.5%)
Pain during sex	29 (44.6%)
Abnormal or heavy menstruation	46 (70.8%)
Infertility	16 (24.6%)
Painful urination during menstruation	21 (32.3%)
Painful bowel movements during menstruation	43 (66.2%)
Other digestive problems (diarrhea, constipation, nausea)	38 (58.5%)
Pain rising over time	33 (50.8%)
Pain, especially excessive menstrual cramps that can be felt	48 (73.8%)
Received treatment for endometriosis	
Hormonotherapy	45 (69.2%)
Fertility treatments	16 (24.6%)
Surgery	25 (38.5%)
Frequency of use of pain killers to manage endometriosis pain	
Never	19 (29.2%)
Only during menstruation	35 (53.8%)
Almost all the time	11 (16.9%)
Level of pain associated with endometriosis	6.63 ± 2.68
Infertility problems due to endometriosis	
No	21 (32.3%)
Yes, I’ve had difficulties in the past	12 (18.5%)
Yes, I still have infertility problems	10 (15.4%)
Not sure	22 (33.8%)

### Comparison of scores between the different treatment options in women with endometriosis

A significantly higher mean depression score was found in women taking hormonotherapy compared to not (5.40 vs. 3.85; p = 0.047). A higher mean anxiety and stress scores was found in those taking fertility treatments vs. not (5.75 vs. 3.96; p = 0.028 and 4.94 vs. 3.82; p = 0.002 respectively). Finally, a lower mean depression (3.88 vs. 5.58; p = 0.021) and anxiety (3.48 vs. 4.98; p = 0.039) scores, as well as a higher mean sexual satisfaction score (91.62 vs. 78.29; p = 0.008) were found in women who underwent surgery compared to not (Table 3).

**Table 3:** Comparison of scores between the different treatment options in women with endometriosis

	Couple satisfaction	Depression	Anxiety	Stress	Perceived partner responsiveness	Sexual satisfaction
Hormonotherapy						
No (N = 20)	15.75 ± 4.09	3.85 ± 2.50	3.80 ± 2.86	3.55 ± 1.79	133.40 ± 32.49	87.93 ± 19.84
Yes (N = 45)	14.04 ± 4.13	5.40 ± 2.99	4.67 ± 2.84	4.33 ± 1.61	119.87 ± 33.09	81.41 ± 20.06
*p*	0.129	**0.047**	0.261	0.085	0.131	0.230
Fertility treatment						
No (N = 49)	15.04 ± 3.92	4.59 ± 3.00	3.96 ± 2.86	3.82 ± 1.80	128.31 ± 31.12	85.62 ± 20.63
Yes (N = 16)	13.13 ± 4.69	5.94 ± 2.46	5.75 ± 2.44	4.94 ± 0.93	110.94 ± 37.07	76.66 ± 17.05
*p*	0.111	0.110	**0.028**	**0.002**	0.069	0.122
Surgery						
No (N = 40)	14.33 ± 4.07	5.58 ± 2.61	4.98 ± 2.77	4.28 ± 1.63	120.68 ± 33.43	78.29 ± 20.16
Yes (N = 25)	14.96 ± 4.36	3.88 ± 3.13	3.48 ± 2.79	3.80 ± 1.78	129.40 ± 32.90	91.62 ± 17.29
*p*	0.554	**0.021**	**0.039**	0.275	0.307	**0.008**

Numbers in bold indicate significant p-values

A significantly higher mean sexual satisfaction score was found in women without endometriosis compared to those with endometriosis (90.83 vs. 83.42; p = 0.006). No significant difference was found in terms of couple satisfaction, depression, anxiety, stress, and perceived partner responsiveness between the two groups (Table 4).

**Table 4:** Comparison of scores between women with and without endometriosis

	Women without endometriosis	Women with endometriosis	*P*
Couple satisfaction	14.84 ± 4.69	14.57 ± 4.16	0.675
Depression	4.51 ± 3.04	4.92 ± 2.92	0.323
Anxiety	4.08 ± 2.95	4.40 ± 2.85	0.432
Stress	3.73 ± 1.97	4.09 ± 1.69	0.136
Perceived partner responsiveness	124.10 ± 31.33	124.03 ± 33.25	0.988
Sexual satisfaction	90.83 ± 19.01	83.42 ± 20.06	**0.006**

Numbers in bold indicate significant p-values

### Bivariate analysis of factors associated with sexual satisfaction

The results of the bivariate analysis of factors associated with sexual satisfaction are displayed in Tables 5 and 6. A higher mean sexual satisfaction score was found in women who did not have children compared to those who did (91.25 vs. 85.91; p = 0.019). Higher couple satisfaction (r = 0.59) and perceived partner responsiveness (r = 0.58) were significantly associated with higher sexual satisfaction, whereas higher depression (r=-0.47), anxiety (r=-0.34), stress (r=-0.33), financial burden (r=-0.15) and number of comorbidities (r=-0.16) were significantly associated with lower sexual satisfaction.

**Table 5:** Bivariate analysis of factors associated with sexual satisfaction

	Sexual satisfaction (mean ± SD)	*P*
Marital status		0.133
Single / divorced	90.99 ± 18.48	
Married	87.71 ± 20.23	
Education level		0.153
Secondary or less	84.48 ± 21.94	
University	89.82 ± 19.13	
Work status		0.149
Unemployed	65.87 ± 11.97	
Employed	62.94 ± 10.53	
Have children		**0.019**
No	91.25 ± 18.33	
Yes	85.91 ± 20.89	

Numbers in bold indicate significant p-values

**Table 6:** Correlation of continuous variables with sexual satisfaction

	1	2	3	4	5	6	7	8	9	10
1. Sexual satisfaction	1									
2. Couple satisfaction	0.59^a^	1								
3. Depression	-0.47^a^	-0.37^a^	1							
4. Anxiety	-0.34^a^	-0.23^a^	0.74^a^	1						
5. Stress	-0.33^a^	-0.29^a^	0.69^a^	0.64^a^	1					
6. Perceived partner responsiveness	0.58^a^	0.75^a^	-0.36^a^	-0.29^a^	-0.30^a^	1				
7. Age	-0.11	-0.08	-0.02	-0.04	-0.01	-0.13^c^	1			
8. Household crowding index	-0.002	-0.11^c^	-0.03	0.01	-0.05	-0.10	-0.09	1		
9. Financial burden	-0.15^b^	-0.11^c^	0.19^b^	0.17^b^	0.12^c^	-0.10	0.01	0.13^c^	1	
10. Average number of comorbidities	-0.16^b^	-0.14^c^	0.20^a^	0.21^a^	0.14^c^	-0.18^b^	0.18^b^	0.19^c^	0.13^c^	1

^a^ p < 0.001; ^b^ p < 0.01; ^c^ p < 0.05; r = Pearson correlation coefficients

### Multivariable analysis of factors associated with sexual satisfaction

The results of the linear regression, taking the sexual satisfaction score as the dependent variable, showed that higher couple satisfaction (Beta = 1.30) and perceived partner responsiveness (Beta = 0.16) were significantly associated with higher sexual satisfaction, whereas higher depression (Beta=-1.70) and having endometriosis compared to not (Beta=-6.51) were significantly associated with lower sexual satisfaction (Table 7).

**Table 7:** Multivariable analysis: Linear regression (ENTER method) using the sexual satisfaction score as the dependent variable

Variable	Unstandardized beta	Standardized beta	*p*	95% CI	VIF
Marital status (married vs. single*)	-2.97	-0.08	0.156	-7.07; 1.14	1.70
Education level (university vs. secondary or less*)	0.33	0.01	0.909	-5.28; 5.93	1.08
Work status (employed vs. unemployed*)	-1.05	-0.03	0.556	-4.56; 2.46	1.16
Having kids (yes vs. no*)	-1.87	-0.05	0.420	-6.43; 2.69	1.93
Diagnosed with endometriosis (yes vs. no*)	-6.51	-0.14	**0.002**	-10.56; -2.47	1.08
Age	0.11	0.04	0.466	-0.19; 0.40	1.69
Household crowding index	1.84	0.05	0.246	-1.28; 4.96	1.18
Financial burden	-0.48	-0.06	0.154	-1.15; 0.18	1.09
Total number of comorbidities	-0.39	-0.02	0.601	-1.87; 1.09	1.21
Couple satisfaction	1.30	0.31	**< 0.001**	0.77; 1.84	2.42
Depression	-1.70	-0.26	**< 0.001**	-2.61; -0.79	3.05
Anxiety	-0.13	-0.02	0.772	-0.98; 0.73	2.50
Stress	0.52	0.05	0.383	-0.66; 1.71	2.07
Perceived partner responsiveness	0.16	0.26	**< 0.001**	0.08; 0.24	2.42

Nagelkerke R^2^ = 48.6%

## Discussion

A significant difference was found between the group of women with endometriosis and the control group in terms of sexual satisfaction. Our results are consistent with evidence provided by the research literature [8, 21, 22], which has shown that symptoms of endometriosis (particularly pain, which was the main concern of participants in our study) affect intimacy and in particular sexual satisfaction. This may be related to the direct association between dyspareunia and decreased sexual satisfaction (higher pain during intercourse, reduced number of intercourses, less satisfying intercourse) [21]. The association between pain and sexual satisfaction could also be explained by the negative expectation cognitive model theory of sexual experiences [8]; this theory states that the experience of pain and the loss of pleasure are recognized recurrently and are reinforced by repeated experiences of pain. Moreover, this association could be due to central sensitization theory; pain in patients with endometriosis can become autonomous and recur, even in the absence of peripheral stimulation [23]. This could imply that the pain associated with the disease, and not necessarily the dyspareunia, can impact the sexual experience of the woman who suffers from it. Interestingly, women with dyspareunia continue to have sex despite the pain. Literature suggests that this may be an attempt by these women to strive to assert themselves in their ideal female image. These findings show that the partner’s pleasure can be more important to many women than their own pleasure and well-being [8]. Future studies are needed to assess the role of the couple’s dynamics in partnered women without marriage in other countries with different cultural backgrounds.

No significant difference was found in terms of perceived partner responsiveness or couple satisfaction between women with and without endometriosis. Our results were similar to those where women perceived their partner as supportive, but felt tension due to the lack of sex in the life of the couple [24]. In one study, some partners of women with endometriosis who expressed very strong emotions such as sad mood, anxiety and even helplessness because of their wife’s endometriosis symptoms were actually able to turn those emotions into acceptance and a stronger bond with the wife, which was beneficial for the couple as a whole [25]. We suggest that this coping strategy could be a helping factor for women with endometriosis in Lebanon. Results from the literature indicate that endometriosis symptoms are associated with symptoms of anxiety and depression [11, 26, 27] and higher levels of stress [16, 28], we found no significant association between endometriosis and signs of depression, anxiety and stress. We suggest that the signs of depression, anxiety and stress found were not significant between the two groups for the following potential reasons: Lebanese women with endometriosis may use more positive coping strategies in order to manage their symptoms. These strategies can be problem-focused, such as knowing more about the illness, self-management, as well as having a strong support system. Emotion-focused coping strategies can include acceptance of illness as well as reliance on spirituality [29, 30].

Moreover, the literature suggests that the heightened risk of psychiatric disorders, such as stress anxiety and depression, is correlated with symptoms of endometriosis, particularly pain symptoms, rather than endometriosis [31]. Therefore, effective management of symptoms of endometriosis may be a protective factor that minimizes the psychological burden among Lebanese patients.

Greater couple satisfaction was significantly associated with greater sexual satisfaction. These results are consistent with findings from the literature indicating that women with lower sexual satisfaction report lower couple satisfaction. Many women report that the lack of sexual activity is jeopardizing their relationships with their partners. In fact, dyspareunia and avoidance of sex have been shown to be common reasons for relationship breakdowns. Moreover, it has been found that the partners of these women tend to feel rejected by the lack of sexual activity [24]. Some partners may also feel the need to take on additional support responsibilities, which can lead them to have emotions of helplessness, frustration, and anger [32].

Greater perceived partner responsiveness was significantly associated with greater sexual satisfaction, these findings are consistent with literature stating that women who perceive their partners as responsiveness tend to have higher scores of sexual function and satisfaction. Genito-pelvic pain is a sexual problem that often negatively affects women who are at risk of it, as well as their partners. The negative consequences of this pain usually include sexual dysfunction and dissatisfaction. However, sexual outcomes have been shown to be influenced by intimacy and partner perception [10]. Assessing the partner’s perception of the situation and comparing the perception of both members would be a good addition to research in the future.

Our results showed significant associations between lower sexual satisfaction scores and higher depression, anxiety, and stress scores, in agreement with the literature [9, 33]. Sexuality is complex; individual psychological, social and physical factors must all be taken into consideration when discussing this topic. Sexual difficulties, especially when due to chronic illness, have a significant impact on a woman’s emotional well-being. Self and body esteem, feelings of femininity, and partner guilt were found to be the main concerns contributing to psychological distress in women with sexual difficulties [8].

### Clinical implications

Through the evaluation of the experience of Lebanese women living with endometriosis and how this disease may affect their emotional state and their intimate relationships, we hope to raise awareness among mental health practitioners, as well as other healthcare providers in charge of these women. This awareness can be a step towards more holistic approaches to treating these patients. This disease has long been considered a purely gynecological problem and treated as such with standardized pharmacological and surgical treatments. However, thanks to the extensive studies over the past decades, we have learned that this condition is not limited to gynecology. The world of research has witnessed accelerating evidence on the complexity of this disease, which translates into different areas of a woman’s life. Holistic and individualized treatment plans appear to be essential for these women, and data from various health fields is therefore needed in order to understand and be able to provide optimal care. In this study, the focus was on the emotional and intimate relational aspects of patients’ lives in hopes of highlighting potential neglected forms of intervention that these women may need to cope with their illness. The exploration of this subject may help us sensitize clinicians on the psychological consequences of the symptoms of endometriosis. The deeper and holistic understanding of the subject could help health care providers consider new, more comprehensive treatment modalities, in addition to purely medical ones, in order to help patients and/or couples. These modalities may include individual counselling or psychotherapy, couple therapy or counselling, sex therapy or counselling etc. Further research is needed to better understand how Lebanese women manage their symptoms of endometriosis and what coping strategies help them in terms of mental health and relational well-being, while taking into account individual medical and psychosocial differences.

### Limitations and strengths

Some limitations may have influenced our results. The subject of sexuality may still be considered taboo by some Lebanese women, even more so by unmarried women, which made it more difficult to find a more meaningful sample. Information bias is present since participants tend not to answer honestly despite the survey being anonymous. A selection bias is present since the sample was collected conveniently and the rate of non-participation is unknown. The Perceived Partner Reactivity Scale (PPRS) and the Women’s Sexual Satisfaction Scales (SSS-W) have not been validated in the Lebanese population. Our questionnaire did not include questions targeting sexual arousal and sexual practices among these women; a more thorough assessment, from a sexological point of view, would have provided us with a clearer and more specific understanding of how women and their sexual life are being affected. Despite the limitations of this study, it has nevertheless strength points; this study is, to our knowledge, the first to study these different variables simultaneously. It should be added that the questionnaire, being disseminated online and through various channels and doctors, reached patients treated by different doctors and by various approaches across Lebanon.

## Conclusion

Endometriosis is a complex chronic disease that poses challenges for both patients and healthcare professionals. Symptoms of endometriosis are varied and can have many implications for women who have them. By analyzing different variables of intimate life, we were able to conclude that there are associations between endometriosis and disturbed sexual satisfaction. This research highlights the need to address these issues simultaneously by professionals caring for patients with endometriosis. By recognizing the complexity of the disease and the diversity of its symptoms, clinicians can target problematic aspects of patients’ lives more optimally. Healthcare professionals need to thoroughly assess all different areas of life when dealing with each case in order to provide adequate treatment, instead of limiting assessments and care to the medical side.

## Data Availability

All data generated or analyzed during this study are not publicly available to maintain the privacy of the individuals’ identities. The dataset supporting the conclusions is available upon request to the corresponding author.
